# Optimization of Welding Parameters Using an Improved Hill-Climbing Algorithm Based on BP Neural Network for Multi-Bead Weld Smoothness Control

**DOI:** 10.3390/ma18174084

**Published:** 2025-08-31

**Authors:** Ying Tong, Guo-Zheng Quan, Hai-Tao Wang, Wei Xiong

**Affiliations:** 1 College of Intelligent Manufacturing and Automotive, Chongqing Polytechnic University of Electronic Technology, Chongqing 401331, China; tongying2004@126.com; 2 Chongqing Key Laboratory of Advanced Mold Intelligent Manufacturing, School of Material Science and Engineering, Chongqing University, Chongqing 400044, China; 3 Key Laboratory of Advanced Reactor Engineering and Safety, Ministry of Education, Collaborative Innovation Center of Advanced Nuclear Energy Technology, Institute of Nuclear and New Energy Technology, Tsinghua University, Beijing 100084, China

**Keywords:** welding parameter optimization, BP neural network, hill-climbing algorithm, weld bead morphology, automated overlay welding, surface smoothness prediction

## Abstract

In multi-pass welding processes, achieving a uniform and smooth weld surface is crucial for mechanical performance and dimensional accuracy. However, the complex nonlinear relationships between welding parameters and weld bead geometry present significant challenges for traditional optimization methods. This study proposes an intelligent prediction and optimization framework that integrates a backpropagation (BP) neural network with an improved hill-climbing algorithm to enhance weld surface smoothness in automated multi-bead overlay welding. Experimental data collected under varying arc voltages, wire feed rates, and welding speeds were used to train the neural network. The improved hill-climbing algorithm adaptively adjusts weights and biases in the BP model to overcome issues of local minima and slow convergence. Comparative results demonstrate that the proposed method significantly outperforms conventional BP approaches in terms of prediction accuracy and convergence efficiency. Furthermore, optimal welding parameters identified by the model yield smoother weld surfaces, reducing the need for post-processing. This work provides a novel solution for intelligent control and real-time optimization in advanced welding systems.

## 1. Introduction

Multi-pass welding is a critical fabrication technique used extensively in the manufacturing of large-scale structures, such as pressure vessels, pipelines, and heavy machinery components [1,2]. Compared to single-pass welding, the multi-pass approach allows for deeper penetration, increased structural integrity, and improved control over material deposition [3]. However, one of the persistent challenges in automated multi-pass welding is the control of weld bead surface morphology—particularly surface flatness—which directly affects the dimensional accuracy, mechanical performance, and post-processing cost of the welded component [4]. Inadequate surface smoothness may lead to residual stress accumulation, dimensional deviations, and increased material removal during subsequent machining operations [5].

Numerous studies have investigated the influence of welding parameters on weld geometry and surface quality [6]. Conventionally, empirical and statistical methods, such as orthogonal design, Taguchi techniques, and response surface methodology (RSM), have been widely adopted to optimize parameters, including arc voltage, welding speed, and wire feed rate. For example, Gyasi et al. [7] employed a full-factorial design to analyze the effects of process parameters on bead width and height in Gas Metal Arc Welding (GMAW), identifying significant interactions between voltage and travel speed. Similarly, domestic researchers have reported the utility of regression-based prediction models for estimating weld bead geometries in automated overlay processes. While such methods provide a general framework for understanding the effects of parameters, their effectiveness is limited when addressing the highly nonlinear and coupled relationships characteristic of multi-pass welding processes [8,9,10].

In recent years, the application of artificial intelligence (AI), particularly neural networks, has garnered attention for its capability to model complex nonlinear systems in welding. Backpropagation (BP) neural networks have demonstrated strong prediction capabilities for weld bead dimensions and surface characteristics [11]. For instance, Zhang et al. [12] applied a BP neural network to predict the width and reinforcement height of single-pass welds with an acceptable accuracy. Liu et al. [13] extended this approach by using BP models to predict mechanical properties of welded joints under varying thermal cycles. Recently, Zhang et al. [14] reported the successful use of a BP neural network to accurately predict weld reinforcement height in MAG welding, further confirming the viability of neural-network-based modeling for welding applications. However, despite their modeling strength, standard BP algorithms are known to suffer from issues such as slow convergence and susceptibility to local minima, particularly when trained on high-dimensional or sparse datasets. This hinders their robustness and generalization in practical welding scenarios.

To overcome these limitations, recent advancements have explored hybrid optimization strategies that integrate neural networks with metaheuristic algorithms, such as genetic algorithms (GA), particle swarm optimization (PSO), and simulated annealing (SA). These combinations have shown improved convergence and predictive performance but often come at the cost of increased computational complexity and parameter-tuning efforts. The hill-climbing algorithm, a simpler yet effective local search technique, offers a promising alternative due to its low computational burden and intuitive implementation [15,16,17]. However, its use in welding parameter optimization remains underexplored, especially when coupled with BP networks for real-time prediction and adjustment [14].

This study proposes a novel hybrid framework that integrates an improved hill-climbing algorithm with a BP neural network to optimize welding parameters for enhanced surface flatness in multi-pass welds. Unlike conventional BP training approaches (i.e., the standard backpropagation algorithm without additional optimization), the proposed method adaptively adjusts the neural network weights by leveraging a hill-climbing strategy to escape local optima and accelerate convergence. The hybrid model is trained using experimental data obtained from flat-plate multi-pass welding trials, where bead geometry was characterized under various combinations of voltage, wire feed rate, and travel speed [18]. The effectiveness of the improved BP model is evaluated in terms of prediction accuracy and computational efficiency, and the resulting optimal process parameters are verified through further experiments [19,20].

Overall, the key contributions of this study are as follows: (1) development of a BP-based prediction model for weld surface flatness in multi-pass welding; (2) incorporation of an enhanced hill-climbing algorithm to improve network convergence and avoid local minima; and (3) identification of optimized process parameters that yield significantly improved weld surface quality. The results demonstrate that the proposed approach provides a reliable and efficient solution for intelligent process control in automated welding systems, with the potential for extension to broader applications, such as additive manufacturing and cladding repair.

## 2. Experimental Methods

### 2.1. Experimental Setup and Equipment

Flat-plate overlay welding experiments were conducted to investigate the relationship between welding process parameters and weld bead geometry. Eight rectangular AISI 1045 steel plates (dimensions 200 mm × 100 mm × 10 mm) were used as the base material. A base plate thickness of 10 mm was selected because it provides a sufficiently thick substrate to prevent full penetration of the weld (plate thickness strongly affects penetration), thereby allowing focus on the surface bead formation without burn-through. A laser welding machine (Model XYZ, Trumpf, Ditzingen, BW, Germany) was used for the experiments. The shielding gas employed was argon (99.99%, Air Products, Allentown, PA, USA). For analysis, MATLAB R2024a (https://www.mathworks.com) and ANSYS 2024 R1 (ANSYS Inc., Canonsburg, PA, USA) were used for data processing and finite element simulations. AISI 1045 steel was selected because it is a commonly used medium-carbon structural steel with well-documented welding characteristics, ensuring that our experimental findings are relevant to typical industrial applications. The filler material was a CN645ACW welding wire with a diameter of 1.2 mm. The chemical compositions of the welding wire and base steel are listed in Table 1 and Table 2, respectively.

**Figure 1:**
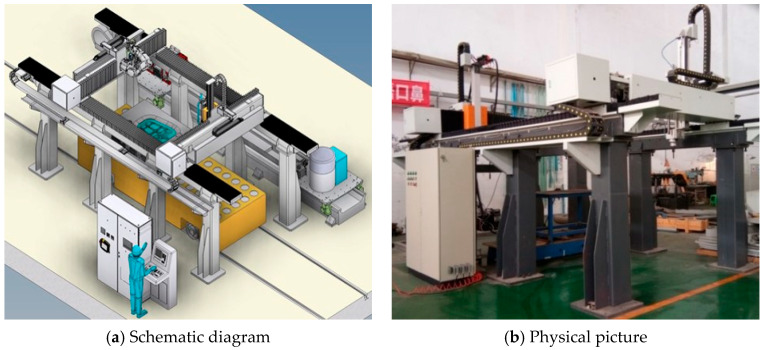
The prototype equipment for material addition by fusing wires.

Welding experiments were conducted using a custom-developed large-scale arc additive manufacturing system, as shown in Figure 1. This system offers a maximum working envelope of 4000 mm by 1600 mm by 600 mm and is capable of accommodating components measuring up to 4650 mm by 1800 mm by 800 mm. The setup includes a power supply, automatic wire feeder, welding torch, and water-cooling unit. The entire welding process was controlled through computer programming to ensure high repeatability and accuracy.

### 2.2. Welding Parameters and Design of Experiments

To evaluate the surface flatness of single-layer multi-pass welds, overlay welding experiments were conducted on AISI 1045 steel plates using the same materials, equipment, and environmental conditions as those employed in the single-pass welding trials. This ensured experimental consistency and allowed for reliable comparison of results.

The selection of welding parameters in this study was based on a backward prediction strategy using a previously established backpropagation (BP) neural network, which was trained to predict weld geometry (i.e., weld width and height) from welding process parameters. Specifically, the BP neural network was first used in an inverse mode to determine suitable combinations of welding voltage, wire feed rate, and welding speed that would produce target weld geometries within predefined practical ranges.

In industrial applications, to ensure high productivity and weld integrity, acceptable weld widths are generally greater than 6 mm, and weld heights exceed 2 mm. Based on this, we constrained our target weld geometry ranges to weld widths between 6 and 10 mm and heights between 2.5 and 3.5 mm, which not only align with industrial standards but also allow for a comprehensive study of the effects of surface flatness.

Nine weld geometry configurations meeting these criteria were selected through inverse prediction. For each configuration, four different weld spacing values (0.6, 0.7, 0.8, and 0.9 times the weld width) were applied, resulting in a total of 36 experimental groups. The corresponding welding parameters—voltage, wire feed rate, and welding speed—are listed in Table 3. In Table 3, the wire feed rate and welding speed values are expressed in mm/min to maintain consistency, where 1 m/min equals 1000 mm/min (for example, 6 m/min = 6000 mm/min). In summary, this inverse prediction approach provided a clear rationale for our experimental design: by ensuring that the chosen welding parameters yield weld bead dimensions within practical industry ranges, we could systematically investigate the effect of varying bead spacing on surface flatness while keeping all test conditions relevant to real-world applications.

**Table 3:** Weld shapes and process parameters used in single-layer multi-pass bead-on-plate welding experiments.

Number	Welding Voltage (V)	Wire Feed Rate (mm/min)	Welding Rate (mm/min)
1	6	2.5	26.1
2	8	2.5	29.1
3	10	2.5	29.6
4	6	3	24.4
5	8	3	26.2
6	10	3	29.3
7	6	3.5	23.6
8	8	3.5	25.6
9	10	3.5	27.8

Note: Wire feed rates (originally in m/min) have been converted to mm/min for consistency (for instance, 6.0 m/min = 6000 mm/min), and welding speeds are also reported in mm/min units. Each configuration (1–9) corresponds to a unique target weld width and height (as detailed in Figure 2), with four different spacing conditions per configuration (spacing values are 0.6, 0.7, 0.8, and 0.9 times the weld width, not listed in this table).

**Figure 2:**
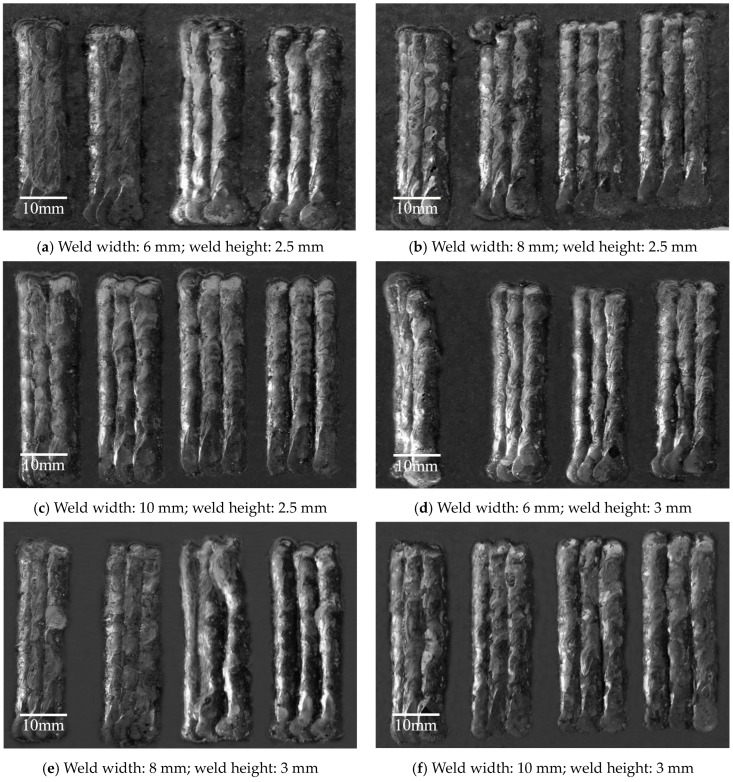

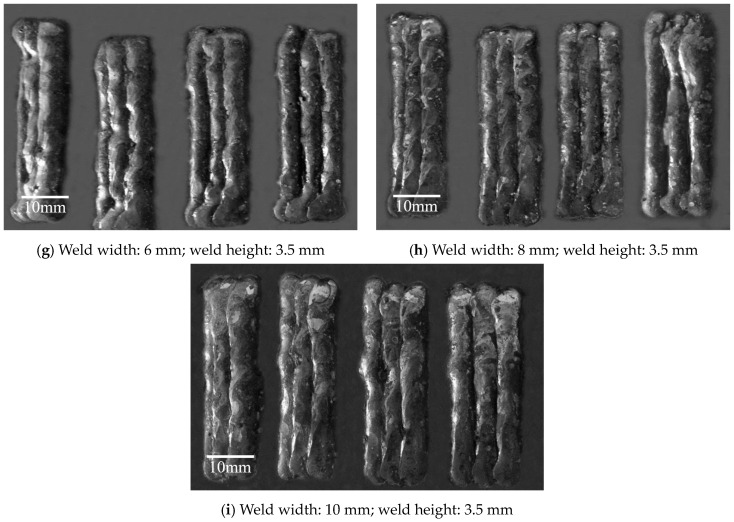
Results of single-layer multi-pass bead-on-plate welding experiments.

The chosen arc voltage range (approximately 24–30 V) in combination with the wire feed rates corresponds to metal transfer modes between short-circuit and spray transfer. At lower voltage settings, metal transfer occurred primarily in the short-circuit mode, whereas higher voltages (~30 V) promoted a more continuous spray transfer. This consideration is important because the mode of metal transfer can influence weld bead geometry, including bead width, reinforcement height, and penetration. In our constant-voltage GMAW system, the wire feed rate effectively controlled the welding current; the selected wire feed rates (up to ~10 m/min, corresponding to a few hundred amperes of welding current) were sufficient to maintain a stable arc and smooth metal transfer without the need for additional current adjustments.

### 2.3. Measurement of Weld Bead Geometry

As shown in Table 3, due to the relatively high wire feed speed used during the welding process, a standardized three-pass welding approach was adopted to mitigate weld shape irregularities caused by wire feeding disturbances and to ensure the reliability of the experimental data.

The experimental results for single-layer multi-pass flat-plate welding are presented in Figure 2, with the experiments arranged sequentially according to the order listed in Table 3. Each image corresponds to a weld geometry and includes four different weld spacing conditions: 0.6, 0.7, 0.8, and 0.9 times the weld width. From a general perspective, when the weld spacing is set to 0.6 times the weld width, the surface of the welds appears relatively smooth and continuous. However, as the spacing increases, the overlapping region between adjacent weld passes becomes progressively smaller, resulting in greater variation in surface flatness. When the spacing reaches 0.9 times the weld width, the overlap between passes is minimal and each weld bead is nearly independent, leading to the poorest surface flatness among all conditions.

In addition, for individual welds with nominal widths of 6 mm, 8 mm, and 10 mm, an observable broadening trend can be seen in the weld profile. This phenomenon indirectly validates the accuracy of the previously developed BP neural network model for single-pass-weld-geometry prediction.

Weld bead geometry was characterized using surface and cross-sectional measurements to evaluate the influence of the process parameters. Bead width and height were measured using a Nikon SMZ745T stereomicroscope (Nikon Corporation, Tokyo, Japan) equipped with a calibrated digital measurement system. Measurements were taken at three transverse positions—40 mm, 50 mm, and 60 mm from one edge of the plate—to minimize local variation. Prior to measurement, specimen surfaces were cleaned to remove contaminants. Observations were performed at a fixed magnification of 7×, and an image analysis software ImageJ, (National Institutes of Health, Bethesda, MD, USA, https://imagej.nih.gov/ij/, accessed on 7 August 2025) integrated with the microscope was used for data processing. The average of the three measurements was used to represent the bead geometry for each condition, forming the basis for training and validating the BP neural network model. For cross-sectional analysis, specimens were sectioned at the same positions using a Sodick VL400Q wire EDM system (Sodick Co., Ltd., Tokyo, Japan) with a cutting accuracy of ±2 μm, employing a 0.25 mm brass wire and deionized water as the dielectric. This method ensured minimal thermal distortion.

**Figure 3:**
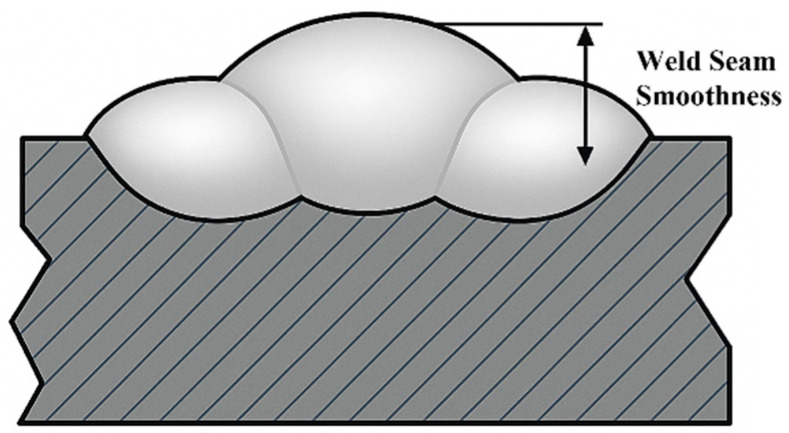
Sketch of single-layer multi-pass welding surface smoothness. Schematic cross-sectional illustration of a single-layer multi-pass weld, showing the weld bead geometry (width and height) and the definition of surface flatness deviation (Δh). (This figure is an illustrative sketch and not an actual experimental result).

Sectioned samples were sequentially ground using 240-, 400, and 600 grit silicon carbide papers under water cooling. This was followed by ultrasonic cleaning with ethanol and subsequent air drying. The cross-sectional features were then examined using the same stereomicroscope. Based on these observations, a schematic of a representative single-pass weld profile was generated and is presented in Figure 3, providing visual support for the analysis of the effects of the process parameters on the weld morphology. The cross-sectional inspection also confirmed that the welds achieved only partial penetration into the 10 mm base plate (no welds completely penetrated). This indicates that the chosen parameters provided sufficient fusion without burn-through, allowing the study to focus on surface bead geometry. Weld penetration was therefore not a primary variable in this work, although it remained within acceptable limits for all experimental runs. Figure 3 is a schematic illustration for visualization purposes and does not depict any specific multi-pass experiment (such as those shown in Figure 2). Instead, it serves to clarify the definitions of weld bead width, weld bead height, and surface flatness used in our analysis.

### 2.4. Data Preprocessing and Normalization

To ensure the accuracy and consistency of statistical results, single-layer multi-pass flat-plate welding experiments were conducted using the same materials, equipment, and experimental conditions as those used in the single-pass welding trials. After eliminating defective results caused by disturbances in wire feeding, surface flatness data were collected using the same measurement procedure.

Following the established protocol, weld surface flatness was measured at three positions along the transverse direction of the plate: 40 mm, 50 mm, and 60 mm from the edge. The evaluation criterion for surface flatness was defined as the vertical difference between the highest and lowest points on the welded surface. This metric effectively reflects the uniformity of the weld profile, with a smaller difference indicating better flatness. The statistical results of surface flatness corresponding to different combinations of weld geometry and spacing are summarized in Table 4.

## 3. BP Neural Network Modeling

### 3.1. Network Architecture Design

To enable an accurate prediction of surface flatness in single-layer multi-pass welds, a backpropagation (BP) neural network model was developed using weld geometry and spacing parameters as input variables. Given that weld shape and weld spacing are the primary factors influencing surface uniformity, the model was constructed with weld width, weld height, and weld spacing as inputs, and weld surface flatness (Δ*h*) as the output response.

A total of 36 experimental datasets were used for model development. Based on statistical sampling, data points with indices 13–16 and 33–36 were selected as the test set (eight samples), while the remaining 28 samples were used as the training set. The normalization of input and output variables, along with the configuration of key network parameters such as transfer functions and learning algorithms, followed the same approach as previously established in the BP neural network model for single-pass weld geometry prediction.

**Figure 4:**
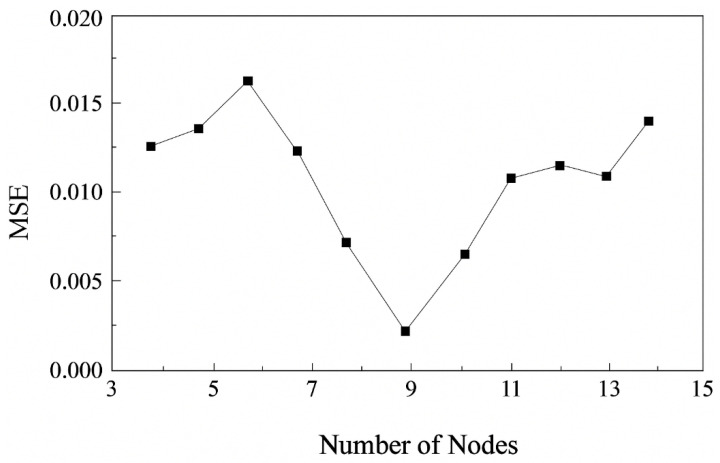
Effect of hidden layer neuron number on BP neural network performance.

Considering that the current model involves only one output variable and that the underlying nonlinear relationships among the variables are relatively moderate, a single hidden layer architecture was adopted. Figure 4 presents the evaluation results of the model performance for different numbers of hidden layer nodes using the mean squared error (MSE) function. The network achieved optimal performance with a minimum MSE when the number of hidden neurons was set to 9. Therefore, the final network structure was determined to consist of one hidden layer with nine nodes, which yielded the lowest MSE and best overall performance. The remaining network configuration parameters, including learning rate, training function, and performance goal, are summarized in Table 5. The BP neural network and optimization algorithm were implemented using MATLAB R2023a (MathWorks, Natick, MA, USA). The network training utilized MATLAB’s Neural Network Toolbox functions, while the improved hill-climbing routine was custom-coded and integrated into the BP training process by the authors.

### 3.2. Training Dataset Construction

To construct a training dataset suitable for predictive modeling of weld surface flatness, three key process parameters were selected based on their dominant influence on the weld morphology: weld width, weld height, and weld spacing. These parameters not only directly affect the geometric formation of single-layer multi-pass welds but also determine the overall surface profile uniformity. Accordingly, they were designated as optimization variables in the response surface methodology (RSM)-based design of experiments for this study.

The dimensionless weld spacing ratio *a* = *λ*/*w* was constrained between 0.6 and 0.9 based on both practical production experience and preliminary single-pass welding trials. When the spacing ratio is below 0.6, excessive bead overlap tends to occur, which can lead to excessive reinforcement, an uneven surface, and potential fusion defects. However, when the ratio exceeds 0.9, the overlap between adjacent beads becomes insufficient, often resulting in surface underfill or visible grooves between weld passes. Therefore, the selected range of 0.6 to 0.9 ensures a balance between adequate bead coverage and acceptable surface flatness. This range also aligns with typical industrial practice for bead-on-plate multi-pass welding processes and conforms to the process window identified in our earlier experimental trials.

A three-factor, three-level design was formulated using a second-order Box–Behnken design (BBD) framework. Each factor was assigned three discrete levels—low, medium, and high—to achieve balanced and efficient coverage of the parameter space. Within the context of practical constraints and weld surface formation characteristics in flat-plate multi-pass surfacing, the parameter ranges were defined as follows: weld width (*w*) from 6 mm to 10 mm, weld height (*h*) from 2.5 mm to 3.5 mm, and weld spacing (*λ*) represented by a dimensionless ratio *a* = *λ*/*w*, where *a* varied between 0.6 and 0.9. This normalization of spacing through ratio *a* allowed a consistent comparative analysis across all experimental conditions.

The complete design matrix is shown in Table 6. The Box–Behnken structure was chosen for its ability to reduce the total number of experimental runs while maintaining sufficient sensitivity to quadratic and interaction effects. Furthermore, the design excludes experimental points where all factors simultaneously assume extreme levels, thereby ensuring the safety, stability, and applicability of all test conditions within the operational window.

Based on the above 17 experimental schemes, the established BP neural network for predicting the surface flatness of single-layer multi-pass welds was employed to compute the target response variable—weld surface flatness (Δ*h*)—for each parameter set. The prediction results are presented in Table 7.

## 4. Performance Metrics and Evaluation

To assess the prediction accuracy and generalization capability of the proposed BP neural network model, a series of quantitative evaluation metrics were employed based on the training and test results [21]. These metrics include the correlation coefficient and the relative mean error, both of which are commonly used to evaluate regression-based neural networks in engineering prediction tasks.

**Figure 5:**
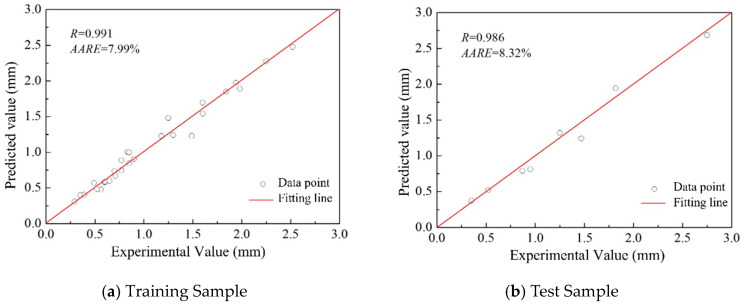
Correlation between the experimental and BP-predicted values **(error bars indicate standard deviation)**.

Figure 5 illustrates the performance evaluation of the BP neural network developed to predict the surface flatness of single-layer multi-pass welds. The correlation coefficients between predicted and actual values are 0.991 for the training set and 0.986 for the test set, indicating a strong positive linear relationship. The relative mean errors are 7.99% for the training data and 8.32% for the test data, respectively. Furthermore, the data points are closely distributed along the fitted line *y* = *x*, which suggests a high prediction accuracy and strong generalization capability of the network.

Overall, these performance metrics demonstrate that the constructed BP neural network is capable of accurately predicting the surface flatness of single-layer multi-pass welds under varying experimental parameters.

## 5. Optimization Method

### 5.1. Standard Hill-Climbing Algorithm Overview

The hill-climbing algorithm is a type of machine learning algorithm classified as a greedy search method. The term “greedy” refers to the approach of solving a globally optimal problem by decomposing it into a sequence of locally optimal subproblems [22]. This is achieved through a top-down iterative process, where each iteration simplifies the overall optimization task into a smaller subproblem, ultimately converging toward a global optimum.

**Figure 6:**
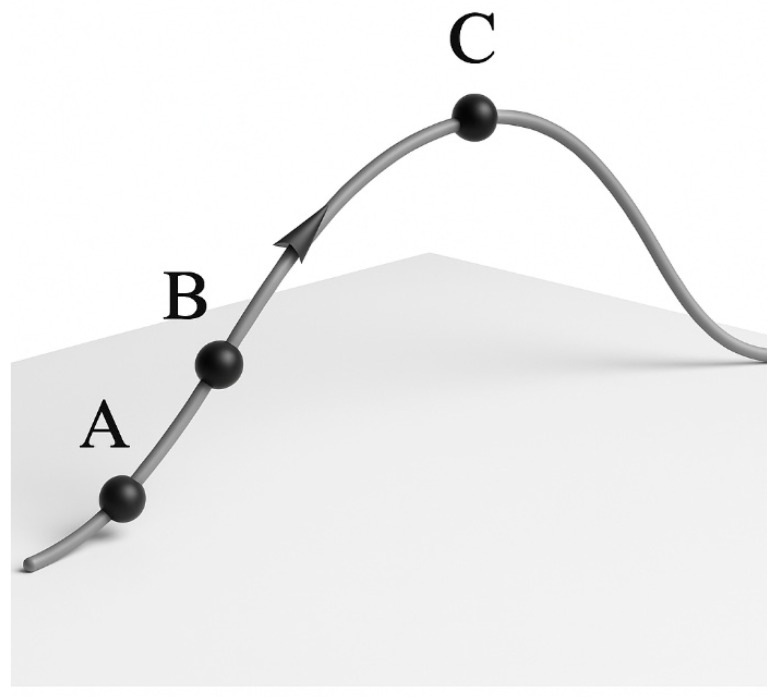
A simple diagram of the hill-climbing algorithm.

In essence, the hill-climbing algorithm iteratively moves in the direction of increasing objective function value, selecting from neighboring states the one with a higher value than the current state as the starting point for the next iteration. If no such neighboring state exists, the algorithm terminates. As illustrated in Figure 6, if the initial point is located at position A, the algorithm identifies a neighboring state B with a higher objective value through one iteration and sets it as the new starting point. This process continues until a higher state C is reached. If the initial point is already at C and no adjacent state offers a higher value, the algorithm terminates and outputs C as the final solution.

In summary, the core logic of the hill-climbing algorithm can be described as follows: the process begins by randomly selecting an initial state *S*_0_ as the current state. If *S*_0_ is already optimal, the algorithm terminates. Otherwise, the algorithm searches among neighboring states for one with a better objective value. If such a state is found, it is assigned as the new current state *S*_0_, and the process is repeated. When no neighboring state offers a better value than the current state, the algorithm concludes and returns the objective value of *S*_0_ as the optimal solution.

### 5.2. Limitations in Welding Parameter Optimization

As discussed in the previous section, the hill-climbing algorithm offers notable advantages for welding parameter optimization. It avoids exhaustive database searches and does not rely on complex parameter tuning, thereby improving both search efficiency and result reliability. However, despite these strengths, the algorithm suffers from several inherent limitations that can impact its performance in practical applications.

First, the tendency to converge to local optima is a significant disadvantage. Since the initial solution *S*_0_ is selected randomly, the algorithm may become trapped in a local extremum if the starting point lies near one, thus failing to reach the global optimum within the defined domain. Second, the algorithm is prone to oscillation near local or global optima, which reduces the convergence rate. This behavior stems from the traditional approach of selecting the first improving neighbor rather than the best among all neighbors, during each iteration. Third, search inefficiency in plateau regions is another limitation. When the objective function presents flat regions, the algorithm cannot determine a clear search direction and thus performs a random walk, leading to inefficiency or premature termination. These limitations have also been noted in previous studies on welding parameter optimization, highlighting the need for hybrid approaches to achieve global optima.

To illustrate these issues, the following test function was defined and used as a benchmark for evaluation:

(1) $$z=3{\left(1-x\right)}^{2}\exp\left(-x^{2}-{\left(y+1\right)}^{2}\right)-10\left(\frac{x}{5}-x^{3}-y^{5}\right)\exp\left(-x^{2}-y^{2}\right)\;-\frac{1}{3}\exp\left(-{\left(x+1\right)}^{2}-y^{2}\right)$$

Equation (1) is a synthetic multimodal function constructed by combining multiple Gaussian-like terms with polynomial expressions to simulate complex objective landscapes. It was designed to contain multiple local extrema, flat plateau regions, and steep ridges, which are commonly encountered challenges in real-world optimization tasks, such as weld surface morphology control. Each component of the function contributes to a specific landscape feature: The first term creates a localized peak with a narrow basin. The second term introduces a more complex region with multiple ripples and steep slopes. The third term adds a broad but shallow feature that simulates a plateau.

Such a structure makes the function ideal for stress testing local search algorithmslike hill climbing. The domain was defined as x ∈ [−3, 3] and y ∈ [−3, 3], encompassing sufficient variation to visualize the convergence behaviors.

**Figure 7:**
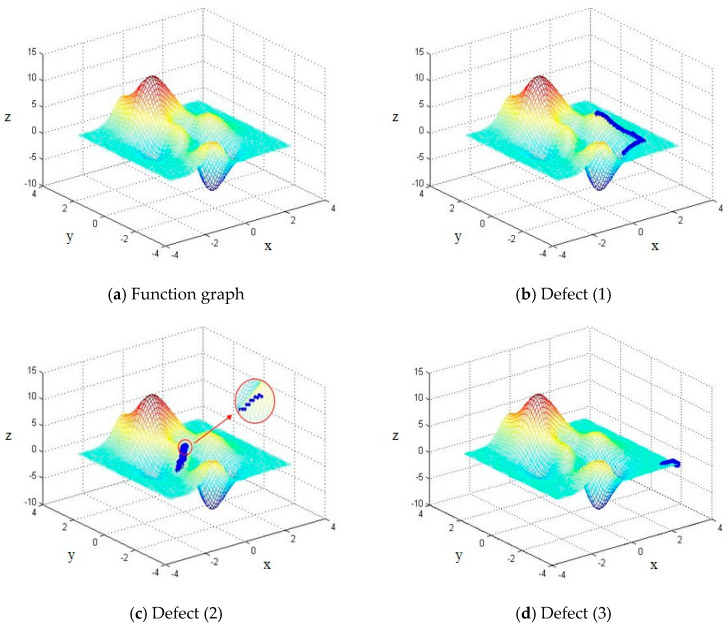
Diagram of hill-climbing algorithm defects.

The hill-climbing paths, visualized as blue dots, are shown in Figure 7. Figure 7a presents a 3D surface plot of the test function, clearly showing a complex landscape with multiple local minima and a plateau near the boundary. In Figure 7b, Limitation 1 is illustrated: the randomly selected starting point falls near a local peak, and the algorithm terminates prematurely with a suboptimal solution. Figure 7c highlights Limitation 2, where oscillations are observed along a ridge, resulting in slow or misdirected convergence. Figure 7d demonstrates Limitation 3, where the search stagnates upon entering a plateau regionand fails to progress toward the global extremum.

These observations highlight the need for algorithmic improvements to overcome the structural shortcomings of the basic hill-climbing approach, especially when applied to nonlinear, multi-extremum optimization problems such as welding parameter tuning.

### 5.3. Improvements to the Hill-Climbing Algorithm

In many practical surfacing welding scenarios, a specific weld bead geometry and surface flatness must be obtained to meet production requirements. Based on the preceding analysis, two backpropagation (BP) neural network models were developed to address this challenge. The first model predicts the geometry of a single weld bead from three input parameters—welding voltage (*x*), wire feed speed (y), and welding speed (z)—and outputs the corresponding weld width (w) and weld height (h). The second model uses the outputs of the first model along with the weld spacing to predict the surface flatness deviation (Δ*h*). Thus, there is a two-stage dependency: the process parameters (*x*, *y*, *z*) influence (w, h), which, along with weld spacing, determines surface flatness.

To unify the optimization process and directly minimize deviation from target weld characteristics, an evaluation function was constructed that indirectly depends on the input parameters (x, y, z) via their influence on the neural network outputs. The function is defined as Equation (2):

(2) $$f\left(x,y,z\right)={\left(W-w\left(x,y,z\right)\right)}^{2}+{\left(H-h\left(x,y,z\right)\right)}^{2}\;+{\left(∆H-∆h\left(x,y,z\right)\right)}^{2}$$

Here, W, H, and ΔH are the target values for the weld width, height, and surface flatness deviation, respectively. The terms *w*(*x*,*y*,*z*), *h*(*x*,*y*,*z*), and *Δh*(*x*,*y*,*z*) represent the predicted outputs of the two neural networks, which are functions of the three input process variables x, y, and z. Therefore, although the equation appears to contain variables *w*, *h*, and *Δh,* they are implicitly dependent on x, y, and z through the neural network models. The minimization of *f* (*x*,*y*,*z*) ensures that the predicted weld characteristics closely match the desired targets.

Considering practical engineering needs, several improvements were made to the conventional hill-climbing algorithm to better optimize Equation (2). To address the first limitation—lack of global convergence—a repeated search strategy was employed. Multiple optimization runs with different random initial states *S*_0_ were performed, and the best result was retained. Trials showed that ten independent runs were sufficient to ensure robustness across 100 optimization trials. To mitigate oscillatory behavior near optima, the algorithm was enhanced to evaluate all neighboring states in each iteration and select the best one, rather than the first that improves the objective. This modification improved convergence speed and solution stability. The third known limitation of hill-climbing—stagnation on plateau regions—was not relevant in this study, as the evaluation function is a smooth quadratic surface with no flat regions in the defined search domain.

### 5.4. Integration with BP Neural Network

**Figure 8:**
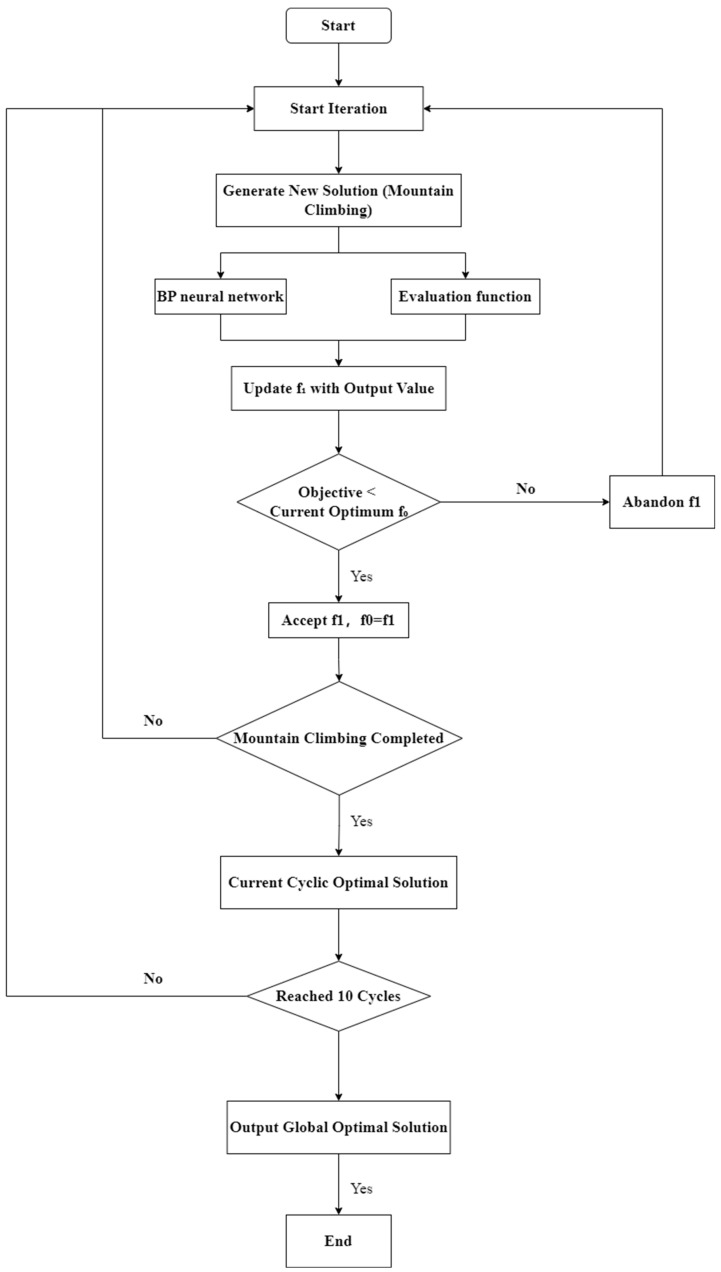
Diagram combining hill-climbing algorithm and BP neural network.

To achieve effective optimization of welding process parameters, an improved hill-climbing algorithm was integrated with a previously developed backpropagation (BP) neural network model, thereby establishing a hybrid optimization framework. As illustrated in Figure 8, the algorithm is initiated by randomly generating an initial solution, which is then input into the objective evaluation function and BP neural network to predict the corresponding weld profile characteristics.

During each iteration, the newly generated solution is evaluated, and its output is compared with the current optimal solution. Based on this comparison, the solution is either accepted or rejected in accordance with the hill-climbing strategy, which is designed to guide the search toward better solutions by iteratively updating candidate parameters. This process continues until a locally optimal solution is identified within one iteration cycle.

To improve the robustness and reliability of the optimization results, the entire search process is repeated for 10 independent runs, each starting from a different randomly selected initial solution. Upon completion, the 10 locally optimal solutions are compared, and the one that yields the minimum value of the objective function is selected as the global-optimal solution. This hybrid approach effectively combines the exploratory power of the hill-climbing algorithm with the predictive accuracy of the BP neural network, thereby enhancing the capability to identify optimal welding parameters under complex nonlinear relationships.

## 6. Results and Discussion

### 6.1. Comparison Between BP and Improved BP Models

In this study, a second-order multivariate regression function was employed to establish a quantitative relationship between three key input variables—weld width (w), weld height (h), and weld spacing ratio (a = λ/w)—and the corresponding response variable, surface flatness deviation (∆h). This modeling approach was based on experimental data obtained from 17 systematically designed test schemes that followed a Box–Behnken design as part of the response surface methodology (RSM). The RSM framework is widely adopted in engineering optimization studies because of its ability to efficiently capture linear, interaction, and quadratic effects among variables using a relatively small number of experiments.

The functional form of the response surface was chosen to be a second-order polynomial due to its flexibility and well-established use in modeling nonlinear behaviors between process inputs and output responses. The regression coefficients were determined through least-squares fitting using the experimental data, ensuring statistical significance of the model. The resulting predictive equation is presented in Equation (3), which is derived from our experimental data:

(3) $$\begin{array}{cc} ∆h=12.1481 & -0.2551\times w-3.17997\times h-22.84483\times a\\ & +0.014206\times w\times h+0.54662\times w\times a\\ & +2.68158\times h\times a-\left(2.92289E-0.03\right)\times w^{2}\\ & +0.2682\times h^{2}+10.01435\times a^{2} \end{array}$$

This regression model captures the main effects, pairwise interactions, and second-order (quadratic) influences of the three input parameters on surface flatness. For example, the negative coefficient of a indicates that increased weld spacing ratio tends to reduce surface flatness, while the significant positive coefficient of *a*^2^ reflects curvature in that effect. The model allows both prediction and optimization of surface flatness across the explored parameter space and serves as the basis for subsequent algorithmic optimization. The fitting quality was validated using R-squared and residual analyses, confirming the model’s suitability for representing the experimental data trends.

To evaluate the performance of the derived response surface model and assess its fitting accuracy, an analysis of variance (ANOVA) and goodness-of-fit test were conducted using Design-Expert software (version 13, Stat-Ease, Inc., Minneapolis, MN, USA, https://www.statease.com/software/design-expert/, accessed on 7 August 2025). As shown in Table 8, the ANOVA results for the response surface model of ∆h are presented. The F-value represents the result of the *F*-test, which is primarily used to determine the statistical significance of the regression relationship between the input variables and the response variable. The mathematical expression for the F-value is provided in Equation (4).

(4) $$F=\frac{\frac{{SS}_{R}}{k}}{\frac{{SS}_{E}}{n-k-1}}$$

In the above equations, k denotes the degrees of freedom of the sample, and *n* represents the number of experimental runs, which is 17 in this study. The regression sum of squares (*SS_R_*) and residual sum of squares (*SS_E_*) for the 17 experimental schemes are calculated as follows: *SS_R_* = ∑(*ŷ − ȳ*)^2^, *SS_E_* = ∑(*y* − *ŷ*)^2^ Here, y represents the observed experimental value, corresponding to the response variable (∆h) in this study; ȳ is the arithmetic mean of the response values; and ŷ is the predicted value from the response surface regression model. Therefore, *SS_R_* reflects the variation in the response variable due to changes in the input variables, while *SS_E_* measures the deviation between the model predictions and the experimental results.

Based on these values, the Design-Expert software automatically calculates the *p*-value corresponding to each *F*-test. A smaller *p*-value indicates a more statistically significant effect of the associated input variable. Generally, a term is considered significant if *p* ≤ 0.05 and highly significant if *p* ≤ 0.01, implying a strong influence on the response variable.

The *p*-value is defined as: $P=P\left(F\geq F_{0}\right)$ where *F*_0_ is the calculated *F*-statistic, and the *p*-value is automatically computed by the software.

The lack-of-fit term represents the proportion of the total error attributable to the model’s inability to explain systematic variations in the data. A large lack-of-fit indicates that the regression model fails to adequately capture the underlying trend of the data.

As shown in Table 8, the *p*-values corresponding to the terms AB, A^2^, and B^2^ are greater than 0.05, indicating that these terms have no statistically significant effect on the response variable (∆h). Therefore, when fitting the functional relationship between the input variables and the response variable, and under the assumption that the interaction effects among input variables are negligible, these non-significant terms were excluded. A revised response surface model for the response variable (∆h) was then re-fitted accordingly. The modified regression equation is presented in Equation (5).

(5) $$\begin{array}{cc} ∆h=9.68189 & -0.25924\times 0.25924\times w-1.45711\times h\\ & -23.04987\times a+0.54662\times w\times a\\ & +2.68158\times h\times a+10.15104\times a^{2} \end{array}$$

The analysis of the revised response surface regression model is shown in Table 9. As shown, the *p*-value for the overall model is less than 0.0001, indicating that the newly fitted regression equation describes a statistically significant relationship between the input variables and the response variable. Furthermore, the *p*-values corresponding to the *F*-tests for all other terms in the table are also significantly less than 0.05, demonstrating that each term included in the revised response surface model is statistically significant. In summary, the updated regression model is considered to be both accurate and reliable, and can be confidently applied in the context of this study.

To determine whether a response surface model can serve as a meaningful approximation of the original experimental data, it is essential to evaluate the goodness of fit of the model to the response values. In this study, the coefficient of determination (R^2^) is introduced as a key metric for assessing the fitting accuracy of the response surface model, as defined in Equation (6)

(6) $$R^{2}=\frac{{SS}_{R}}{{SS}_{T}}$$

In this equation, *SS_R_* denotes the regression sum of squares, and *SS_T_* represents the total sum of squares. Similar to the correlation coefficient *R* used in the performance evaluation of the previously discussed BP neural network, the coefficient of determination *R^2^* ranges from 0 to 1. A value of *R*^2^ approaching 1 indicates a higher accuracy of the response surface model, suggesting that the predicted values closely match the experimental data. Conversely, an *R*^2^ value near 0 implies a more scattered distribution of sample points and a weaker correlation between input and response variables.

However, the use of *R*^2^ as a measure of model-fitting accuracy can be influenced by the number of input variables. When the number of variables is small and all are included in the model, the *R*^2^ value may be artificially inflated, leading to an overestimation of the model’s performance. To address this, the adjusted coefficient of determination $R_{adj}^{2}$ is introduced. This statistic accounts for the number of input variables and provides a more accurate assessment of the fitting performance of the response surface model, as shown in Equation (7).

(7) $$R_{adj}^{2}=1-\frac{\frac{{SS}_{E}}{n-k-1}}{\frac{{SS}_{T}}{n-1}}$$

where *SS_E_* denotes the residual sum of squares, *SS_T_* is the total sum of squares, *n* represents the number of experimental runs (17 in this study), and *k* is the number of degrees of freedom. The closer the adjusted coefficient of determination ($R_{adj}^{2}$) is to the unadjusted coefficient (*R*^2^), and the closer both are to 1, the more strongly it suggests that the relationship between the input and response variables is significant and necessary in the response surface model. In this study, the response surface model for the surface flatness (∆h) of single-layer multi-pass welds yields a coefficient of determination R^2^ = 0.9922, indicating a strong correlation between the predicted and experimental values. The adjusted coefficient of determination $R_{adj}^{2}$ = 0.9876 further confirms the high fitting accuracy and reliability of the developed model.

To further evaluate the accuracy and validity of the response surface model, a normal probability plot of the studentized residuals was used to assess its statistical characteristics. The expression for the studentized residuals is given in Equation (8):

(8) ei*=ei1−hijσa2+b2=c2

where, $e_{i}^{*}$ denotes the studentized residual, $e_{i}$ is the ordinary residual, $\hat{\sigma }$ represents the estimated standard error, and $h_{ij}$ is the leverage (the diagonal element of the hat matrix). It can be seen that the studentized residual is derived from the ordinary residual by applying studentization, which removes the influence of both measurement units and leverage on the residuals. If the points in the normal probability plot of the studentized residuals form an approximately straight line, it can be concluded that the residuals follow a normal distribution.

**Figure 9:**
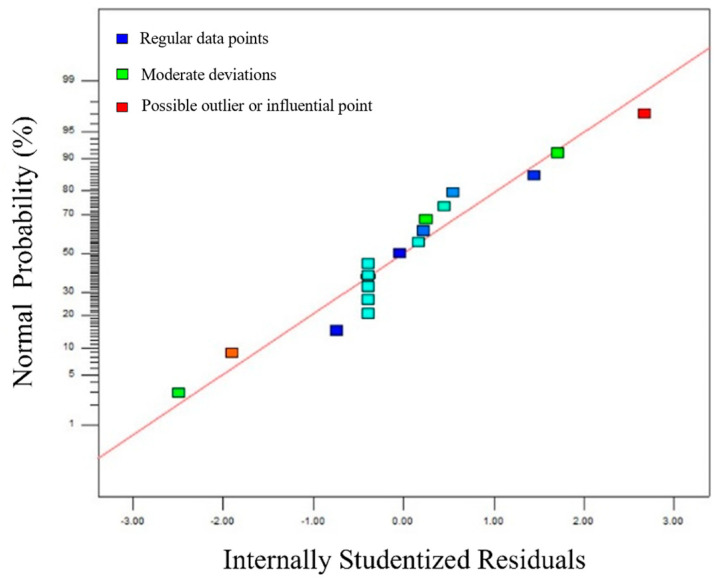
Normal probability plot of the Studentized Residuals for the response surface model.

Figure 9 presents the normal probability plot of the studentized residuals for the response surface model of surface flatness (∆h) in single-layer multi-pass welds. As shown in the figure, all data points lie closely along a straight line, indicating that the response surface model is statistically accurate and valid.

**Figure 10:**
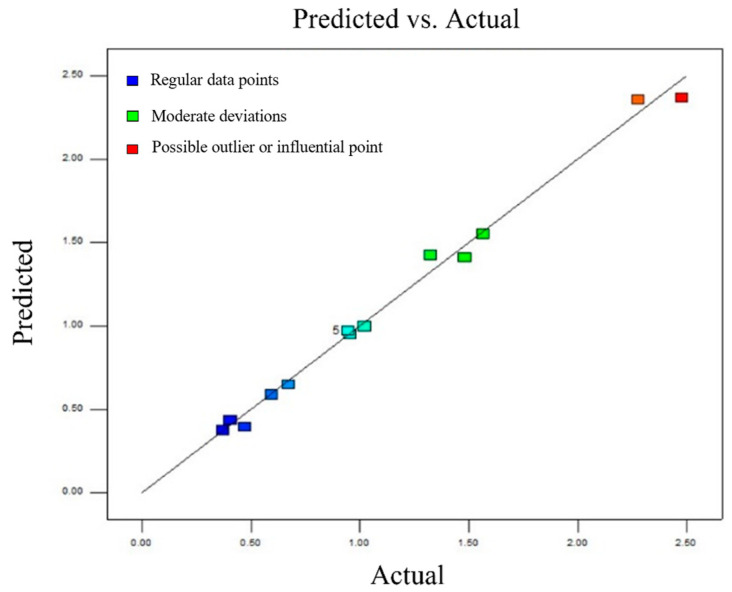
Comparison of the predicted value by the response surface model and the value based on the BP neural network.

Figure 10 shows a comparison between the predicted values obtained from the response surface model developed in this study and those predicted by the BP neural network. It can be observed that all data points are closely clustered around the reference line (*y* = *x*), indicating a high level of agreement between the two models. This demonstrates that the response surface model reliably captures the relationship between the input variables and the response.

**Figure 11:**
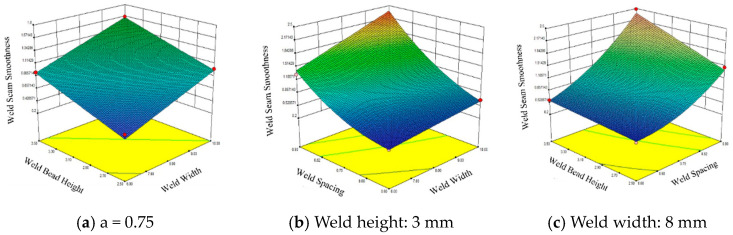
Response surface of single-layer multi-pass welding surface smoothness.

Figure 11 presents the response surface of surface flatness (∆h) for single-layer multi-pass welds. The results indicate that when the weld spacing is held constant, the surface flatness increases with both weld width and weld height, and this increasing trend remains generally consistent across the examined range. When the weld height is fixed, surface flatness increases with both weld width and weld spacing. Notably, the influence of weld spacing becomes increasingly significant as it increases, whereas the effect of weld width on surface flatness remains relatively uniform. Furthermore, when the weld width is kept constant, surface flatness also increases with weld height and weld spacing. The overall trends observed in this case are similar to those in the previous two scenarios. These findings suggest that all three parameters—weld width, weld height, and weld spacing—exert a positive and cumulative influence on the surface flatness of multi-pass welds.

### 6.2. Influence of Welding Parameters on Weld Bead Geometry

#### 6.2.1. Effect of Weld Width on Surface Smoothness

Under a constant weld height, an increase in the weld width leads to a flatter profile of a single weld bead, potentially facilitating a more uniform surface in multi-pass welding. However, cross-sectional observations reveal that the overall surface unevenness does not decrease accordingly. In contrast, when the weld spacing ratio and weld height remain constant, the surface flatness of the weld increases significantly with the weld width, indicating that wider welds may result in greater surface irregularities in multi-pass welds.

**Figure 12:**
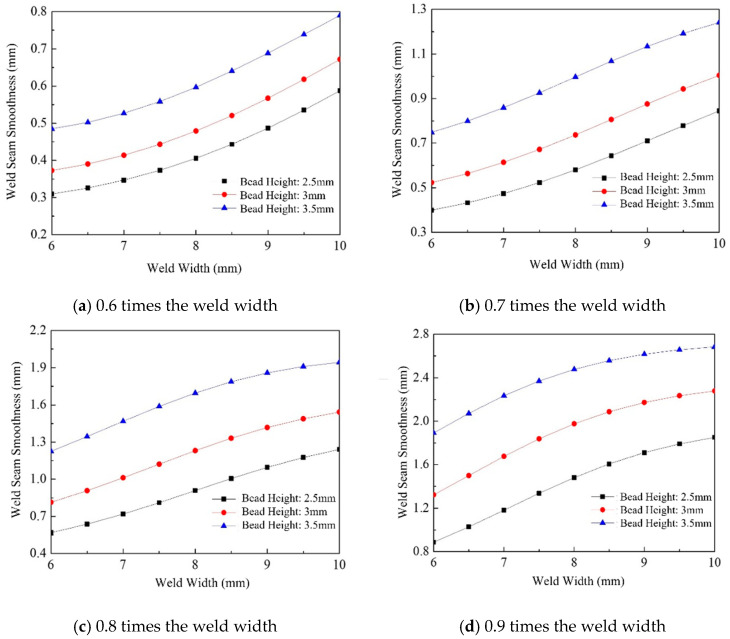
Effect of weld width on welding surface smoothness.

Figure 12 illustrates the trend of weld surface flatness as a function of weld width. The flatness variation ranges from 0.31 mm to 2.68 mm, and overall, it increases with weld width. Under the same weld width and spacing conditions, the surface flatness is primarily determined by the height of a single weld bead; a greater weld height results in higher flatness values. Comparing Figure 12a–d, it is evident that the increasing trend in surface flatness becomes less pronounced as the weld spacing ratio increases. Specifically, for spacing ratios of 0.6 and 0.7, the rate of increase in surface flatness is more significant, whereas for ratios of 0.8 and 0.9, the trend becomes progressively more moderate.

This behavior can be attributed to two factors. First, at lower weld spacing ratios, the surface flatness values deviate further from the height of a single weld bead. As the weld width increases, the actual spacing under a constant ratio also increases, resulting in a marked increase in flatness. Second, lower spacing ratios cause greater overlap between adjacent weld beads, leading to a significant filler metal accumulation. This accumulation increases the peak height of the multi-pass weld relative to that of a single pass, thereby increasing the overall surface drop. In contrast, with higher spacing ratios, the overlap area is smaller, and the filler accumulation mainly affects the lowest regions of the weld surface, thus reducing the extent of surface unevenness.

#### 6.2.2. Effect of Weld Height on Surface Smoothness

The height of a single weld bead directly influences the smoothness of the weld profile; a greater weld height results in a steeper weld contour, making it more challenging to achieve a smooth surface in multi-pass welding. In this section, based on the previously established BP neural network model for predicting the surface flatness of single-layer multi-pass welds, the weld surface flatness values were predicted for weld heights ranging from 2.5 mm to 3.5 mm, at 0.125 mm intervals. The analysis was conducted for weld widths of 6, 8, and 10 mm and weld spacing-to-width ratios of 0.6, 0.7, 0.8, and 0.9.

**Figure 13:**
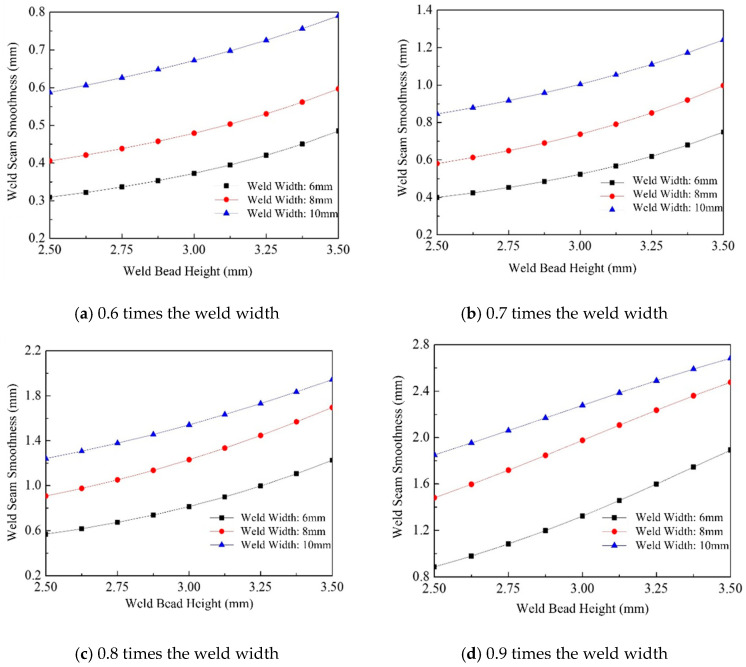
Effect of weld height on welding surface smoothness.

As shown in Figure 13, under the same weld width and spacing conditions, the surface flatness increases with increasing weld height. Unlike the effect of weld width on surface flatness, the influence of the weld spacing ratio exhibits a progressively stronger trend with increasing weld height. This is primarily due to the filler metal accumulation effect that occurs under fixed weld spacing ratio conditions. At lower weld heights, this accumulation has a more pronounced impact on the surface flatness of multi-pass welds, whereas its influence diminishes as the weld height increases.

#### 6.2.3. Effect of Overlap Ratio on Weld Surface Uniformity

**Figure 14:**
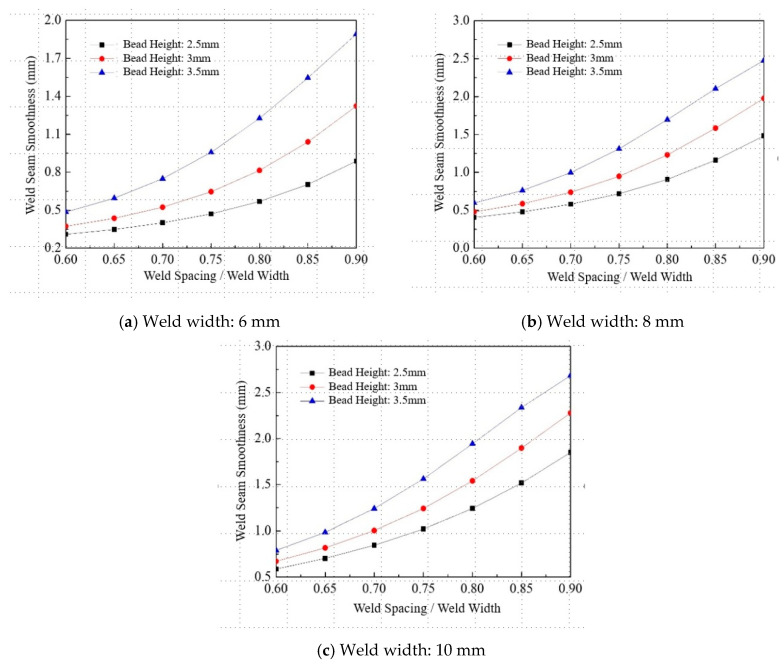
Effect of weld spacing on welding surface smoothness.

Figure 14 shows the trend of weld surface flatness with respect to weld spacing during multi-pass welding. The predictions were performed using increments of 0.05 times the weld width as the spacing step, under weld widths of 6 mm, 8 mm, and 10 mm, and weld heights of 2.5 mm, 3.0 mm, and 3.5 mm. The results indicate that, under fixed weld width and height, smaller weld spacings lead to greater overlaps between adjacent weld passes, resulting in increased filler metal accumulation at the intersection zones. This accumulation contributes to a smoother overall weld surface.

As the weld spacing ratio increases, the surface flatness deteriorates more significantly. The beneficial effect of filler accumulation in reducing surface undulation becomes increasingly insufficient to compensate for the increasing surface irregularity caused by larger spacing.

Integrating the findings from earlier analyses on the effects of weld width and height, it can be concluded that a larger weld width, greater weld height, and increased weld spacing collectively lead to higher weld surface flatness values, indicating poorer surface uniformity. This is attributed to two main factors: (1) wider welds result in larger absolute spacing under the same spacing ratio, thereby increasing the geometric disparity across the weld surface, and (2) greater weld heights inherently increase the vertical profile variation, exacerbating surface unevenness in multi-pass welds.

### 6.3. Optimization Results and Process Parameter Recommendations

The response surface model developed in this study has been thoroughly validated using multiple verification methods from different analytical perspectives. The model has proven to be both accurate and reliable within the parameter range defined by the experimental design. However, to ensure its applicability in real-world production environments, incorporating actual manufacturing constraints into the optimization process is essential.

In the specific production scenario addressed in this work, a mechanical hammering operation is applied after each single-layer weld pass to mitigate residual welding stress. This operation utilizes a pneumatic hammer driven by compressed air at a pressure of 0.5 MPa to deliver impacts to the weld surface. Empirical measurements indicate that this post-weld treatment consistently reduces the weld height by approximately 0.5 mm.

To ensure a smooth and uniform surface profile after hammering, the target surface flatness for single-layer multi-pass welds was set to 0.5 mm. Based on this requirement, the weld geometry parameters were optimized. A subset of the optimization results is presented in Table 10, which includes various combinations of weld width (w), weld height (h), and weld spacing (a) that meet the post-hammering surface flatness constraint (∆h ≈ 0.5 mm).

Among the candidate parameter sets, the 7th configuration was selected due to its favorable balance between weld quality and production efficiency. This set features a weld width of 7.99 mm, a pre-hammering weld height of 2.5 mm, and an inter-bead spacing of approximately 5.35 mm, resulting in a post-hammering flatness deviation of 0.51 mm, which is within the acceptable tolerance.

By applying inverse prediction through a previously trained backpropagation (BP) neural network tailored for weld profile modeling, the optimal process parameters were determined to be a welding voltage of 30.3 V, a wire feed speed of 12,600 mm/min, and a welding speed of 950 mm/min, effectively ensuring the desired weld geometry is achieved.

To validate the predicted optimal parameter set, we conducted an additional welding experiment using these parameters (arc voltage: 30.3 V, wire feed speed: 12,600 mm/min, and welding speed: 950 mm/min). The weld produced under these conditions was measured after the standard hammering treatment and exhibited a surface flatness deviation of approximately 0.5 mm, matching the model’s prediction. This successful validation confirms that the optimized parameter combination is practically achievable and effective, even though the required voltage and wire feed speed slightly exceed the initial experimental range, ensuring that the welding process remains efficient and stable under these settings.

## 7. Conclusions

This study presents a hybrid optimization approach that combines a backpropagation (BP) neural network with an improved hill-climbing algorithm to address the challenges of weld bead geometry prediction and surface smoothness optimization in automated multi-pass overlay welding. The main conclusions drawn from this research are as follows:

(1) A robust dataset was established through comprehensive welding experiments that varied arc voltage, wire feed rate, and welding speed. The resulting weld bead geometries, including width, height, and spacing, provided a reliable foundation for predictive modeling.
(2) The BP neural network model effectively captured the nonlinear mapping between welding parameters and weld bead morphology. However, the original BP algorithm suffers from convergence inefficiency and susceptibility to local minima, limiting its optimization capability.
(3) The improved hill-climbing algorithm, incorporating adaptive step sizing and neighborhood perturbation, successfully enhances the training process of the BP neural network. The hybrid model exhibited faster convergence, lower prediction error, and superior generalization when compared to the conventional BP approach.
(4) Optimization results showed that the best welding surface smoothness was achieved with a relatively high arc voltage (≈30 V), a high wire feed rate (~13,000 mm/min), and a moderate welding speed (~900 mm/min), demonstrating the practical utility of the proposed method in guiding process selection.
(5) The developed model provides a practical and intelligent tool for real-time parameter tuning in automated welding systems, contributing to higher quality control and reduced post-processing needs.

## Figures and Tables

**Table 1:** Chemical composition of welding wire (wt/%).

C	Si	Mn	Cr	Mo	S	P	Others
<0.25	<0.6	<0.8	4.0–7.5	1.0–3.0	<0.012	<0.015	2.0–3.0

**Table 2:** Chemical composition of 45 carbon steel (wt/%).

C	Si	Mn	P	S	Cr	Ni	Cu	Fe
0.42–0.5	0.17–0.37	0.5–0.8	≦0.035	≦0.035	≦0.25	≦0.25	≦0.25	Bal.

**Table 4:** Statistical results of single-layer multi-pass welding surface smoothness.

Serial Number	Weld Width (mm)	Weld Height (mm)	Weld Spacing (mm)	Surface Flatness (mm)
1	6	2.5	3.6	0.29
2	6	2.5	4.2	0.35
3	6	2.5	4.8	0.49
4	6	2.5	5.4	0.77
5	8	2.5	4.8	0.39
6	8	2.5	5.6	0.6
7	8	2.5	6.4	0.9
8	8	2.5	7.2	1.25
9	10	2.5	7	0.6
10	10	2.5	8	0.85
11	10	2.5	9	1.3
12	10	2.5	10	1.84
13	6	3	3.6	0.35
14	6	3	4.2	0.52
15	6	3	4.8	0.95
16	6	3	5.4	1.05
17	8	3	4.8	0.56
18	8	3	5.6	0.7
19	8	3	6.4	1.49
20	8	3	7.2	1.94
21	10	3	7	0.71
22	10	3	8	0.83
23	10	3	9	1.6
24	10	3	10	2.25
25	6	3.5	3.6	0.52
26	6	3.5	4.2	0.77
27	6	3.5	4.8	1.18
28	6	3.5	5.4	1.98
29	8	3.5	4.8	0.65
30	8	3.5	5.6	0.85
31	8	3.5	6.4	1.6
32	8	3.5	7.2	2.52
33	10	3.5	7	0.87
34	10	3.5	8	1.25
35	10	3.5	9	1.82
36	10	3.5	10	2.75

**Table 5:** Parameter setting of the BP neural network.

Parameter Name	Parameter Values
Number of hidden layers	1
Number of the hidden layer nodes	9
Maximum number of training sessions	1000
Minimum gradient requirement	1 × 10^−20^
Learning efficiency	0.01
Adjustment parameter	0.005
Training requires accuracy	0.0001

**Table 6:** Box−Behnken experimental design with three factors and three levels.

Experimental Number	*w* (mm)	*h* (mm)	*a*
1	8	3	0.75
2	10	2.5	0.75
3	6	3	0.9
4	8	3.5	0.9
5	8	3	0.75
6	10	3	0.6
7	8	3	0.75
8	8	3	0.75
9	8	2.5	0.9
10	6	3	0.6
11	10	3	0.9
12	10	3.5	0.75
13	8	2.5	0.6
14	8	3.5	0.6
15	6	2.5	0.75
16	8	3	0.75
17	6	3.5	0.75

**Table 7:** Experimental plan and target response variable values.

Experimental Number	Input Variables			Output Variable
	*w* (mm)	*h* (mm)	*a*	△*h* (mm)
1	8	3	0.75	0.95
2	10	2.5	0.75	1.02
3	6	3	0.9	1.32
4	8	3.5	0.9	2.48
5	8	3	0.75	0.95
6	10	3	0.6	0.67
7	8	3	0.75	0.95
8	8	3	0.75	0.95
9	8	2.5	0.9	1.48
10	6	3	0.6	0.37
11	10	3	0.9	2.28
12	10	3.5	0.75	1.56
13	8	2.5	0.6	0.41
14	8	3.5	0.6	0.6
15	6	2.5	0.75	0.47
16	8	3	0.75	0.95
17	6	3.5	0.75	0.96

**Table 8:** ANOVA for response surface model of ∆h.

Source Data	Sum of Squares	Degrees of Freedom	Mean Square	F-Value	*p*-Value
Model	5.65	9	0.63	182.17	<0.0001
A-*w*	0.73	1	0.73	210.92	<0.0001
B-*h*	0.61	1	0.61	178.15	<0.0001
C-*a*	3.80	1	3.80	1102.43	<0.0001
AB	8.073 × 10^−4^	1	8.073 × 10^−4^	0.23	0.6432
AC	0.11	1	0.11	31.21	0.0008
BC	0.16	1	0.16	46.94	0.0002
A^2^	5.755 × 10^−4^	1	5.755 × 10^−4^	0.17	0.6950
B^2^	0.019	1	0.019	5.49	0.0516
C^2^	0.21	1	0.21	62.03	0.0001
Residual	0.024	7	3.447 × 10^−3^		
Lack of Fit	0.024	3	8.042 × 10^−3^		

**Table 9:** ANOVA for new response surface model of ∆h.

Source Data	Sum of Squares	Degrees of Freedom	Mean Square	F-Value	*p*-Value
Model	5.63	6	0.94	212.59	<0.0001
A-*w*	0.73	1	0.73	164.68	<0.0001
B-*h*	0.61	1	0.61	139.09	<0.0001
C-*a*	3.80	1	3.80	860.72	<0.0001
AC	0.11	1	0.11	24.37	0.0006
BC	0.16	1	0.16	36.65	0.0001
C^2^	0.22	1	0.22	50.05	<0.0001
Residual	0.044	10	4.414 × 10^−3^		
Lack of Fit	0.044	6	7.357 × 10^−3^		

**Table 10:** Optimization results of welding seam parameters.

Number	*w* (mm)	*h* (mm)	*a*	△*h* (mm)
1	6	3.47	0.63	0.5
2	6	3.29	0.65	0.5
3	6	2.5	0.79	0.5
4	6	2.6	0.77	0.5
5	6.05	3.5	0.62	0.5
6	6.31	3.5	0.61	0.5
7	7.99	2.5	0.67	0.51

## Data Availability

The original contributions presented in this study are included in the article. Further inquiries can be directed to the corresponding authors.
